# Response to “Association between health literacy and behaviors among shift workers: an observational cross-sectional study with mediation analysis”

**DOI:** 10.1093/joccuh/uiag009

**Published:** 2026-02-13

**Authors:** I Wayan Gede Suarjana, Hanifa M Denny

**Affiliations:** Doctoral Program of Public Health, Faculty of Public Health, Universitas Diponegoro, Semarang, Central Java 50275, Indonesia; Department of Public Health, Faculty of Sport Science and Public Health, Manado State University, Manado, North Sulawesi 95618, Indonesia; Faculty of Public Health, Universitas Diponegoro, Semarang, Central Java 50275, Indonesia

Dear Editor

We commend Morikawa et al^1^ for their well-designed study examining the mediating role of health literacy in the association between shift work and health behaviors. By applying mediation analysis to a large sample of manual workers, the authors address an important gap in the occupational health literature. Nevertheless, we believe that the principal conclusion, namely that health literacy does not mediate the relationship between shift work and health behaviors, warrants a more careful interpretation when examined through evidence derived from Indonesia.

In Indonesia, health literacy rarely functions as an independent individual attribute. Instead, it frequently serves as a marker of broader structural inequality that reflects educational attainment, employment security, access to health insurance, and the availability of occupational health services.^2^ National surveys and workforce studies consistently show that shift workers with low health literacy are simultaneously exposed to adverse working conditions, including long working hours, unstable contracts, and limited access to workplace health promotion.^3^ Under such circumstances, health literacy is strongly embedded within antecedent structural determinants that constrain behavioral choice.

From this perspective, the absence of a mediating effect of health literacy does not necessarily indicate that health literacy is irrelevant to health behavior formation. Rather, it suggests that health literacy may lack the analytical independence required to function as a mediator in statistical models. When structural constraints dominate daily decision making, individual cognitive capacity to access or interpret health information has limited opportunity to translate into behavioral change. As a result, mediation models that treat health literacy as a freely operating variable may underestimate its true role in shaping health behaviors among shift workers.^4^

Another consideration relates to population characteristics. Studies conducted among formal sector workers in Indonesia indicate that workplace health programs can elevate health literacy to a minimum uniform level across employees. However, such improvements are often not accompanied by proportional changes in smoking behavior, diet, or physical activity.^5^ This phenomenon may compress the variability of health literacy scores, thereby reducing the statistical power to detect mediation effects. In this situation, null mediation findings may reflect limited variance rather than the absence of a meaningful relationship.

Furthermore, the mediation framework employed in the study assumes that health behaviors arise primarily from rational information-based decision processes. Evidence from Indonesia suggests that health behaviors among shift workers are more strongly shaped by adaptation to work schedules, physical fatigue, and time scarcity than by deliberate health information processing. Smoking, irregular eating patterns, and low physical activity frequently function as coping responses to occupational demands rather than as outcomes of inadequate knowledge. This behavioral mechanism further limits the capacity of health literacy to operate as a direct mediator.

In conclusion, we suggest that the findings reported by Morikawa et al do not negate the importance of health literacy, but instead point to its role as an indicator of structural inequality rather than a standalone causal pathway. Recognizing health literacy as structurally embedded may advance theoretical understanding and prevent policy interpretations that undervalue health literacy interventions among shift workers. For countries such as Indonesia, strategies that integrate health literacy improvement with changes in working conditions and employment security are likely to be more effective in reducing health behavior disparities.
